# Vaccination with autologous dendritic cells loaded with autologous tumor lysate or homogenate combined with immunomodulating radiotherapy and/or preleukapheresis IFN-α in patients with metastatic melanoma: a randomised “proof-of-principle” phase II study

**DOI:** 10.1186/1479-5876-12-209

**Published:** 2014-07-22

**Authors:** Francesco de Rosa, Laura Ridolfi, Ruggero Ridolfi, Giorgia Gentili, Linda Valmorri, Oriana Nanni, Massimiliano Petrini, Laura Fiammenghi, Anna Maria Granato, Valentina Ancarani, Elena Pancisi, Valentina Soldati, Serena Cassan, Angela Riccobon, Elisabetta Parisi, Antonino Romeo, Livia Turci, Massimo Guidoboni

**Affiliations:** 1 Immunotherapy Unit, Istituto Scientifico Romagnolo per lo Studio e la Cura dei, Tumori (IRST) IRCCS, Meldola, FC, Italy; 2 Unit of Biostatistics and Clinical Trials, Istituto Scientifico Romagnolo per lo Studio e la Cura dei Tumori (IRST) IRCCS, Meldola, Italy; 3 Radiotherapy Unit, Istituto Scientifico Romagnolo per lo Studio e la Cura dei Tumori (IRST) IRCCS, Meldola, Italy; 4 Center for Biological Resources, Istituto Scientifico Romagnolo per lo Studio e la Cura dei Tumori (IRST) IRCCS, MeldolaFC, Italy

**Keywords:** Vaccine, Melanoma, Radiotherapy, Dendritic cell

## Abstract

**Background:**

Vaccination with dendritic cells (DC) loaded with tumor antigens elicits tumor-specific immune responses capable of killing cancer cells without inducing meaningful side-effects. Patients with advanced melanoma enrolled onto our phase II clinical studies have been treated with autologous DC loaded with autologous tumor lysate/homogenate matured with a cytokine cocktail, showing a clinical benefit (PR + SD) in 55.5% of evaluable cases to date. The beneficial effects of the vaccine were mainly restricted to patients who developed vaccine-specific immune response after treatment. However, immunological responses were only induced in about two-thirds of patients, and treatments aimed at improving immunological responsiveness to the vaccine are needed.

**Methods/Design:**

This is a phase II, “proof-of-principle”, randomized, open-label trial of vaccination with autologous DC loaded with tumor lysate or homogenate in metastatic melanoma patients combined with immunomodulating RT and/or preleukapheresis IFN-α. All patients will receive four bi-weekly doses of the vaccine during the induction phase and monthly doses thereafter for up to a maximum of 14 vaccinations or until confirmed progression. Patients will be randomized to receive:

(1.) three daily doses of 8 Gy up to 12 Gy radiotherapy delivered to one non-index metastatic field between vaccine doses 1 and 2 and, optionally, between doses 7 and 8, using IMRT-IMAT techniques;

(2.) daily 3 MU subcutaneous IFN-α for 7 days before leukapheresis;

(3.) both 1 and 2;

(4.) neither 1 nor 2.

At least six patients eligible for treatment will be enrolled per arm. Daily 3 MU IL-2 will be administered subcutaneously for 5 days starting from the second day after each vaccine dose. Serial DTH testing and blood sampling to evaluate treatment-induced immune response will be performed. Objective response will be evaluated according to immune-related response criteria (irRC).

**Discussion:**

Based upon the emerging role of radiotherapy as an immunologic modifier, we designed a randomized phase II trial adding radiotherapy and/or preleukapheresis IFN-α to our DC vaccine in metastatic melanoma patients. Our aim was to find the best combination of complementary interventions to enhance anti-tumor response induced by DC vaccination, which could ultimately lead to better survival and milder toxicity.

## Background and rationale

### Malignant melanoma

Malignant melanoma represents one of the most challenging problems in modern oncology. Although it accounts for only 4% of incident cases of skin cancers, it is responsible for more than 80% of all skin cancer deaths [1]. Moreover, its incidence has dramatically increased in recent decades [2]. Whilst early stage melanoma (i.e. primary thin melanomas without regional lymph node or distance involvement) has a very favourable prognosis with a recurrence rate of less than 10% at five years, patients with advanced disease experience a very poor clinical course, with median survival ranging from 4 to 12 months [3]. Furthermore, until very recently, the clinical efficacy of the available treatments was disappointing. In early 2011, the FDA approved the clinical use of ipilimumab, a CTLA 4-blocking monoclonal antibody which, in addition to significantly improving the survival of patients with metastatic melanoma, primarily demonstrated the effectiveness of anti-cancer therapy based only upon immune system targeting [4,5]. However, ipilimumab causes a generalized activation of the immune system which can, in extreme cases, induce severe autoimmune side-effects [6]. On this basis, immunotherapeutic approaches capable of eliciting effective anti-tumor immune responses without the related autoimmunity must be sought developed.

### Dendritic cell vaccination

Vaccination with dendritic cells (DC) loaded with tumor antigens has been shown to elicit tumor-specific immune responses potentially capable of killing cancer cells without inducing significant side-effects [7]. DC are widely distributed antigen-presenting cells that play a central role in the activation and regulation of the immune response [8]. In particular, DC determine the final outcome of adaptive immune responses against a specific antigen, *i.e*. whether it will be tolerated or whether a specific immune response will be triggered against it. The final outcome depends on the balance between different signals acting on the DC and DC antigen uptake. Indeed, if immature DC recruited to peripheral tissues find appropriate “danger signals” (*i.e*. pathogen products capable of triggering toll-like receptors), they undergo maturation and migrate to lymph nodes where they initiate a specific immune response. Conversely, if danger signals are not present, or if concurrent immunosuppressive stimuli occur, DC maturation does not take place, thus inducing immune tolerance against the antigens they are presenting [8]. As cancer progresses, tumor cells acquire the ability to evade the immune response by selecting lesser immunogenic variants (“cancer immunoediting”) [9,10] and/or by producing immunosuppressive cytokines and other biologically active substances that strongly influence the ability of DC to prime and sustain effective immune responses [11,12].

In 1996, Schadendorf’s group was the first to test, in melanoma patients, the feasibility of a vaccination strategy aimed at reconditioning DC function by their differentiation and loading with tumor antigens *ex vivo*, thus permitting the effects of a DC-tolerant tumor microenvironment to be overcome [13]. Since this first experience, it has been estimated that over 1,000 patients with different tumors have been treated using different starting cells and differentiation/maturation protocols, as well as different antigen sources and administration routes [14].

Since 2001, patients with advanced melanoma enrolled onto our phase II clinical studies have been administered autologous DC loaded with autologous tumor lysate/homogenate matured with a cytokine cocktail, showing a clinical benefit (PR + SD) in 55.5% of evaluable cases [15,16].

Like de Vries et al. [17], we saw that patients who developed anti-tumor immunity after vaccination experienced a better clinical outcome [16,18]. In particular, we observed that patients developing delayed type hypersensitivity (DTH) against autologous tumor lysate or keyhole lympet hemocyanin (KLH) after at least four courses of the vaccine showed a median overall survival (OS) of 22.9 months compared to the 4.8 months of DTH-negative cases (Log-rank test, p = 0.007) [16]. Intriguingly, both the disease control rate (DCR) and OS obtained with DC vaccination considerably exceeded those obtained with ipilimumab in a similar patient setting [4]. However, although differentiation and maturation conditions used for the production of DC vaccines have been standardized, only two-thirds of patients develop potentially effective anti-tumor immune responses. We recently reported that patients showing upregulated expression of tumor endothelial marker-8 (TEM-8) in monocyte-derived DC upon maturation do not develop immune reactivity against autologous tumor lysate (ATL) or KLH on DTH and experience a very poor clinical outcome after DC vaccination [19].

TEM-8 is an integrin-like cell surface protein specifically expressed by embryonic and tumor endothelial cells (not, however, in the adult endothelium) whose functions are still not fully understood [20]. It was recently shown that TEM-8 is upregulated in tumor-associated endothelial cells after exposure to interleukin-1β (IL-1β) [21]; interestingly, IL-1β, together with tumor necrosis factor-α (TNF-α) and other cytokines produced by tumor cells (melanoma included) [22], can negatively affect the differentiation of monocyte-derived DC in vitro [23]. As IL-1β and TNF-α are components of the cytokine maturation cocktail currently used to prepare the DC vaccine, it would not be surprising that, at least in a subset of patients, these cytokines support the differentiation of DC toward an immunosuppressive TEM-8 + phenotype rather than the more immunogenic and clinically active phenotype. In light of this, new treatment conditions capable of preventing such an occurrence should be identified.

### Potential effects of Interferon-α (IFN-α) on circulating precursors of DC

IFN-α is a cytokine belonging to type-I interferons that is currently approved for clinical use in B-cell non-Hodgkin’s lymphoma, advanced renal cell cancer and, in the adjuvant setting, malignant melanoma. This cytokine is secreted by virtually all cells after challenge with viral or bacterial products, but it can also be produced by transformed cells or by normal cells under mitogenic stimulus. Its effects on tumor or virus-infected cells are pleiotropic, ranging from the direct inhibition of proliferation to the suppression of oncogene expression and the induction of tumor suppressor genes. Moreover, IFN-α positively affects the activity of both the adaptive and innate immune system, and enhances immune recognition of tumor cells [24]. Furthermore, IFN-α inhibits the expression of the proangiogenic inflammatory cytokine, interleukin-8 (IL-8), and IL-1β [25], both of which induce an immunosuppressive phenotype in monocyte-derived DC [23].

It is tempting to speculate that treatment with IFN-α, whilst inhibiting the expression of IL-1β, may also suppress the maturation-induced TEM-8 upregulation of DC observed in non-immunoresponsive patients, thus revertingthe TEM-8 associated immunosuppressive phenotype. In partial support of this hypothesis is our observation of positive immunization to DTH in all patients who underwent therapy with IFN-α a maximum of 30 days before starting DC vaccination (our unpublished data), suggesting that IFN-α priming performed *in vivo* before leukapheresis may enhance the immunostimulatory profile of DC. Moreover, IFN-α priming may also have a “mobilizing” activity on DC precursors: it was recently reported that 1–3 MU subcutaneous IFN-α enhances the proportion of circulating CD14+ and CD14 + CD16+ monocytes in both healthy donors and melanoma patients [26].

In the light of these findings, administration of IFN-α before leukapheresis may positively modulate the immunological and clinical efficacy of DC vaccination.

In particular, preemptive IFN-α should:

– (1.) lead to the production of more highly immunogenic monocyte-derived DC;

– (2.) mobilize peripheral DC precursors, thus enhancing leukapheresis yields;

– (3.) positively modulate the immunogenicity of melanoma cells *in vivo*.

### Immunomodulating effects of radiotherapy

Ionizing radiation therapy (RT) is known for its capacity to kill cancer cells and other cells within the tumor stroma, including endothelial cells and intratumoral lymphocytes [27]. Although tumor cells killed by RT may represent a suitable source of antigens for DC uptake and presentation to T cells, it is widely accepted that optimal activation of T cells by DC can be achieved only in the presence of inflammatory or “danger” signals. On this basis, the hypothesis that exposure to ionising radiation (IR) generates a proinflammatory “danger” microenvironment supports claims made of a strong synergy between radiotherapy and immunotherapy. In particular, IR induces the secretion of the pro-inflammatory cytokines IL-1β and TNF-α in both animal cancer models and cancer patients [28,29]. Moreover, IR upregulates the expression of Fas/CD95 receptor and NKG2D-L in cancer cells [30], encouraging the recognition and killing of the “altered self” and MHC-I and co-stimulatory molecules, which leads to a more accurate recognition by immune effectors [31]. Finally, RT can facilitate the homing of both antigen-presenting and effector T cells to the tumor bed by eliciting inflammatory signals and changes in extracellular matrix proteins and by inducing the expression of adhesion molecules by endothelial cells in the tumor microenvironment [32,33]. Demaria S. *et al*. showed that irradiation of a tumor xenograft in a murine model protected against rechallenge from the same tumor cells outside the irradiation field. This anti-tumor effect was probably the result of a specific immune response as it is not observed in immunodeficient nude mice. It would seem to be tumor-type specific *i.e*. (growth of a second graft of a different cell line is not affected by irradiation of the first graft) and is synergistic with administration of the Flt-3 (FMS-like tyrosine kinase 3) ligand. Flt-3 alone (no RT) has no effect on the second graft, probably because of a nonspecific immunity boost provided by the Flt-3 ligand. Overall, these data suggest that RT delivered to a tumor site may also prime the immune system against related tumor cells at some distance from the irradiation field (“abscopal effect”) and that, in the absence of additional immune system stimulation, the radiation-induced immune response has no clinical effect. Accordingly, the combined use of DC vaccination and RT with “immunological” intent should theoretically strengthen the effect of the vaccine by acting as an *in situ* boost in which tumor antigens are released and captured by intratumoral DC in a microenvironment positively conditioned by ionising radiation [34-37].

### Description of the investigational product

Since 2001, IRST Somatic Cell Therapy Laboratory has produced an advanced medicinal product in the form of a therapeutic vaccine composed of autologous DC pulsed with autologous tumor lysate or homogenate for patients with metastatic melanoma or kidney cancer [15,16,38-40]. The vaccine can be administered to patients either immediately after preparation or after thawing of cryopreserved aliquots. Details on manufacturing methods are provided in Additional file 1.

### Freshly-prepared vaccine

Each vaccine dose is prepared from patients’ monocytes obtained by leukapheresis. After leukapheresis, a part of the monocytes obtained are cultured and the remainder is cryopreserved in aliquots to be used for the manufacture of subsequent vaccine doses. Monocytes are cultured for six days in serum-free, GMP (Good Manufacturing Practice) certified medium supplemented with granulocyte-macrophage colony-stimulating factor (GM-CSF) and interleukin-4 (IL-4) to obtain immature dendritic cells (iDC). These immature DC are pulsed with autologous lysate or homogenate prepared from surgically removed metastatic lesions. After pulsing, DC are then matured for 48 hours in the presence of a cytokine cocktail (TNF-α, IL-1β, IL6, and PGE2). Mature DC (mDC) are then collected, washed, counted and re-suspended in sterile saline (total 7–15 × 10^6^ cells) for immediate intradermal administration to patients.

### Cryopreserved vaccine

The vaccine is produced from the whole leukapheresis product according to the previously described protocol. After the maturation step, pulsed mDC are collected, washed, counted, re-suspended in sterile saline, aliquoted (total 7–15 ×10^6^ cells) and cryopreserved by automated freezing. Before administration, the mDC are thawed, washed, re-suspended in saline and immediately injected intradermally into patients.

### Delayed-type hypersensitivity test (DTH)

DTH testing is a classic method for measuring cellular immune reactivity. This technique involves the intradermal administration of an antigen preparation and the monitoring of the degree of erythema and induration produced 24–48 hours after injection. The response reflects antigen-specific recruitment and the activation of CD4+ to release T-helper 1 cytokines (in particular, IFN-α) and CD8+ effector T cells into the injection site [41,42]. In our experience, as in that of other groups, a positive response to the DTH test performed with soluble antigen (in our studies with ATL) after vaccination with DC in metastatic melanoma patients was strongly correlated with a better clinical outcome [15-18]. DTH testing using another antigen, KLH, has been studied and its positivity has also been found to be associated with improved clinical outcome in vaccinated patients. We use KLH as an immunological adjuvant in the preparation of our DC vaccine and have seen that positive DHT testing to this protein is a reliable indicator of immunologic competence in vaccinated patients. DTH testing does not require extensive training or the use of costly equipment and can easily be performed at the patient’s bedside. All these features make it a feasible, low-cost immunomonitoring method that permits the evaluation of immunologic efficacy in a clinical trial setting.

### Trial design and statistical considerations

This is a phase II, randomized, open-label trial aimed at assessing whether different external immunostimulant conditions, *i.e*. preleukapheresis IFN-α and external RT to one target lesion, enhance, alone or in combination, the immunological efficacy of autologous DC loaded with tumor lysate or homogenate in metastatic melanoma patients. The design follows the concept of “proof-of-principle” studies [43,44] in which the rationale for a given treatment or treatment combination is explored by analyzing disease and/or treatment-specific biological parameters. The study has been approved by the Local Ethics Committee (ClinicalTrails.gov identifier: NCT01973322) and will be carried out in compliance with the Helsinki declaration.

Therapeutic cancer vaccines are a heterogeneous group of complex biologics with distinctly different clinical characteristics which require the development of new clinical paradigms. Hoos *et al*. proposed the use of a “proof-of-principle” trial which would include patients in a metastatic setting without rapidly progressive disease to allow vaccines to have sufficient time to induce biological activity and which would also incorporate immune and molecular markers.

Our objectives include the demonstration of biological activity as proof-of-principle, defined as any effect of the vaccine combination on the target disease using biological markers as study endpoints (immunological response). We also aim to identify which, if any, external immunostimulant conditions give the best result in terms of biological activity, *i.e*. improving the ability of the DC vaccination to induce clinically effective anti-tumor immune responses. We chose the randomized design to select the most promising regimen for further evaluations (phase II or phase III trials) [45,46]. This approach, based upon the assumption that immunotherapy is only expected to be effective in patients showing efficient induction of anti-tumor immune responses (“targeted endpoint”), allows us to reduce the number of patients required to evaluate the potential efficacy of an experimental treatment.

Randomization guarantees the avoidance of selection bias, excluding the preferential inclusion of patients in certain treatment arms. This approach, whilst not identifying which factors contribute to immunogenicity or which regimens are significantly better than others, will, however, highlight a treatment or treatment combination that warrants further investigation. The sample size per treatment must be large enough to ensure, with strong probability, that if one treatment or treatment combination is superior to all others by a specific amount, then it will have the largest sample mean and will therefore be selected. In particular, if one of the external immunostimulant conditions investigated shows higher clinical activity than that observed in the other conditions by a predetermined amount, that condition will be superior to the others with a given probability and will merit further clinical investigation. By enrolling six patients in each treatment arm, we will obtain a 90% probability of correctly selecting the best investigated external immunostimulant condition if the superior arm, if present, has a true expected outcome that is at least 15% higher in terms of irDCR (immune-related disease control rate) than that of the inferior arms [46]. Otherwise, the immunological primary endpoint (immunological efficacy) will guide the choice of treatment combination to further develop using the same method. If a treatment or a combination of treatments is not identified as superior, the vaccine regimen (without external immunostimulant conditions) will be considered for further investigation.

### Primary objectives

#### Clinical objective

To select the regimen that has the best irDCR defined as the proportion of subjects with an immune-related best overall response (irBOR) of confirmed immune-related complete response (irCR), immune-related partial response (irPR) or immune-related stable disease (irSD). The irDCR will be compared in the different treatment arms to identify possible differences in clinical activity between the different external immunostimulant conditions combined with the autologous tumor lysate-loaded DC vaccine.

#### Immunologic objective

The immunologic efficacy of the different external immunostimulant conditions used in combination with the autologous tumor lysate-loaded DC vaccine will be assessed by DTH development against KLH and tumor homogenate and also by IFN-γ-ELISPOT analysis of circulating effectors specific for tumor antigens known to be expressed in melanoma, as described in the “Immunologic endpoint assessment” section.

### Secondary objectives

#### Clinical objectives

To define further the clinical efficacy of the different treatment arms, including an analysis of OS (as indicated by median survival and survival rates at 1- and 2-year follow-ups), immune-related time to progression (irTTP), immune-related overall response rate (irORR), immune-related duration of response (irDOR), immune-related time to response (irTTR) and immune-related progression-free survival (irPFS). All time-dependent clinical endpoints (irTTP, OS) will be estimated using the Kaplan-Meier method and the log rank test will be performed on differences in these endpoints across the treatment arms. Frequency tables will be created for all categorical variables. Continuous variables will be presented using mean and standard deviation or median and range. Given the explorative intent of the study and the limited sample size, we are aware that we may be exposed to a high level of false positive results. Unless otherwise indicated, the analysis of demography and baseline characteristics will be performed on all randomized patients. Demographic and laboratory results will be summarized using descriptive statistics.

#### Immunologic objectives

Secondary objectives also include the evaluation of the effects of preleukapheresis IFN-α on DC yield and potency, and of maturation-induced TEM8 expression on DC:

a) DC yield: the number of DC obtained per ml of blood processed by leukapheresis will be recorded at each leukapheresis and compared between the different treatment arms (IFN-α vs no-IFN-α);

b) DC potency: DC vaccine potency will be analyzed using an ELISPOT-based version of the COSTIM assay which will evaluate the co-stimulatory ability of dendritic cells;

c) TEM-8 upregulation observed upon DC maturation in non-immunoresponsive patients will be evaluated by qRT-PCR and the distribution of TEM-8 mRNA iDC: mDC ratio will be compared between the different treatment arms (IFN-α *vs* no-IFN-α). The percentage of patients in each treatment group reporting an adverse event (AE) up to 30 days after vaccination will be tabulated with 95% confidence intervals according to type of AE. The overall rate of grade 3–4 AEs will be calculated on the basis of treatment group. In addition, summary statistics of clinically significant laboratory abnormalities will be tabulated.

### Overview of study phases

#### Screening

**Figure 1:**
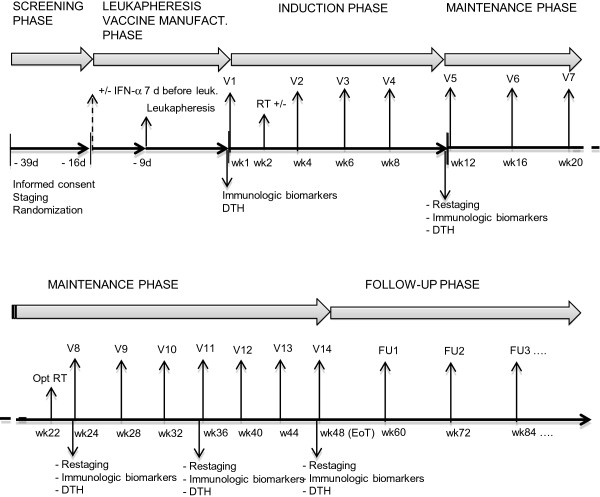
Induction and maintenance phase schedule.

Before entering the study, patients will be required to undergo tumor tissue collection according to Good Manufacturing Practice (GMP) guidelines, as per current Standard Operating Procedures of the IRST Laboratory of Somatic Cell Therapy (STC Lab) (Figure 1). Tumor tissue will be stored in the Therapeutic Cell Bank of the STC Lab until use for vaccine manufacturing. Only patients from whom autologous tumor tissue has been collected by the STC Lab under GMP will be eligible for the study. After informed consent has been obtained, patients will enter the screening phase (tumor staging and clinical and laboratory assessments) to determine their eligibility for the trial. In particular, patients must have at least one measurable and one non-measurable lesion.

#### Leukapheresis and vaccine manufacturing

At the end of the screening phase, patients will be randomized and will enter the leukapheresis and vaccine manufacturing phase. Each patient will be assigned to one of the following arms:

(1.) three daily doses of 8 Gy - 12 Gy radiotherapy delivered to one non-index metastatic field (optional to one additional field) between vaccine doses 1 and 2 and between doses 7 and 8, using IMRT-IMAT techniques;

(2.) daily 3 MU subcutaneous IFN-α for 7 days before leukapheresis;

(3.) both 1 and 2 external immunostimulant conditions;

(4.) neither 1 nor 2 external immunostimulant conditions.

At least six patients eligible for treatment will be enrolled per arm.

#### Induction

After vaccine preparation, the patients will start treatment in the induction phase. Before the first dose of the vaccine (day 1), patients will undergo blood sampling for immunological markers (quantification of circulating tumor-specific immune effectors) and the baseline DTH test. Three MU IL-2 will be administered subcutaneously daily for five days starting from the second day after each vaccine dose. Vaccine doses will be given intradermally in two sites close to inguinal or axillary lymph node stations that have not been the site of previous surgical exeresis. The first dose (wk1) will consist of the freshly prepared vaccine, whereas cryopreserved aliquots will be used for all other doses. The second dose will be administered after three weeks (wk4) to allow for the completion of quality assurance/quality control (QA/QC) assessments on the cryopreserved vaccine batches that will be used across all further phases. The remaining two doses of the induction phase will be administered after a 2-week interval (wk6 and wk8). At wk2, patients randomized to receive RT will undergo the planned treatment: one non-measurable (or non-index measurable, if available) lesion will be irradiated using IMRT-IMAT techniques to facilitate tumor antigen release and and antigen uptake by intratumoral DC under “danger” conditions created by ionising radiation, thus potentially boosting immunization induced by the DC vaccine. The remaining measurable lesion (s) will be used as an index lesion (s) to evaluate objective response according to irCR.. The induction phase will end with tumor restaging, blood sampling for immunological biomarkers and DTH test (the day before wk12 vaccine dose).

#### Maintenance

Patients showing irCR, irPR or irSD or non-confirmed irPD will enter the maintenance phase during which they will receive further vaccine doses at four-week intervals until irPD is confirmed, for up to a maximum of 14 vaccinations (wk48). Tumor assessments, blood sampling for immunological biomarkers and DTH tests will be performed every 12 weeks (wk24, 36 and 48).

In the event of vaccine shortage, patients will undergo additional leukapheresis at least two weeks after the last RT (according to treatment arm) and at least one week before the next vaccine dose: patients assigned to the IFN-α arms will repeat this treatment for seven days before leukapheresis with the same schedule and dosage. Patients undergoing additional leukapheresis must have negative tests for HBV, HCV, HIV and treponema not dating back more than 30 days. This procedure will be performed to facilitate the completion of the QA/QC assessment on the new cryopreserved vaccine batch. At week 22 (between vaccine doses V7 and V8), patients randomized to RT can optionally repeat this treatment on a non-index, not previously irradiated lesion, if available. On the basis of irCR, patients showing irPD will continue treatment until confirmation of progression. Patients with confirmed irPD showing positive DTH may, at the physician’s discretion, continue treatment until the next tumor assessment. If, at the next re-staging, further progression is observed, these patients will be discontinued.

### Study population

Patients with unresectable stage III or stage IV melanoma with at least two measurable lesions who may or may not have undergone previous lines of specific therapy (i.e. chemotherapy, immunotherapy, targeted therapy, etc.).

#### Inclusion criteria

(1.) Signed written informed consent: patients must be willing and able to give written informed consent and this must be provided before starting the screening procedure;

(2.) Availability of autologous tumor tissue that fulfils acceptance criteria

(3.) Patients must have histologically or cytologically confirmed malignant unresectable stage III or stage IV melanoma (any type considered);

(4.) Patients must have a minimum of two lesions, one of which must be accurately measurable in two perpendicular dimensions (at least one diameter >20 mm and the other dimension >10 mm) using or at least 10 × 10 mm using spiral CT scan;

(5.) Patients carrying BRAF mutation-positive melanoma must have received previous vemurafenib unless they are not eligible for or have refused treatment;

(6.) Patients treated with previous first-line therapy must have received ipilimumab unless they are not eligible for or have refused treatment;

(7.) Pre-treated brain metastases that have been clinically stable for at least six months and that do not require corticosteroids are allowed;

(8.) ECOG performance status 0–1;

(9.) Negative screening tests for HIV, HBV, HCV and syphilis not dating back more than 30 days before any of the GMP-regulated activities required are performed (leukapheresis, collection of tumor biopsies to be used for tumor lysate/homogenate preparation);

(10.) Prior lines of chemotherapy, immunotherapy or biological therapy (e.g. inhibitors of BRAF or c-Kit, ipilimumab, etc.) for advanced disease are allowed (patients must have completed prior treatments at least four weeks before the first vaccine dose);

(11.) Males and females aged 18–70 years;

(12.) Females of childbearing potential (WOCBP) must use an adequate method of contraception to avoid pregnancy throughout the study and for eight weeks after the study to minimize the risk of pregnancy;

(13.) Patients must have normal organ and marrow function as defined below:

– leukocytes >1,500/μL;

– absolute neutrophil count >1,000/μL;

– platelets >80,000/μL;

– total bilirubin ≤ 2 × ULN;

– AST (SGOT)/ALT (SGPT) <2.5 × ULN;

– creatinine ≤ 2 mg/dl.

#### Exclusion criteria

Participants will not be eligible for the study if ANY of the following apply:

(1.) Patients with positive tests for HCV, HBV, HIV or syphilis (specific blood testing must be performed no more than 30 days prior to any GMP regulated activity (leukapheresis and collection of tumor biopsies to be used for tumor lysate/homogenate preparation);

(2.) Patients with unresectable or metastatic BRAF V600 mutation-positive melanoma eligible for vemurafenib may not be enrolled for first-line treatment;

(3.) Patients eligible for ipilimumab treatment can only be given the drug as first-line treatment;

(4.) Patients who have had chemotherapy or radiotherapy in the four weeks prior to entering the study or those who have not recovered from adverse events caused by treatment administered more than four weeks earlier;

(5.) Participation in another clinical trial with any investigational agent in the 30 days prior to study screening;

(6.) Patients with known progressing and/or symptomatic brain metastases;

(7.) Uncontrolled intercurrent illness including, but not limited to, ongoing or active infection, symptomatic congestive heart failure, unstable angina pectoris, cardiac arrhythmia or psychiatric illness/problems in social situations that would limit compliance with study requirements (at the physician’s discretion);

(8.) Other known malignant diseases in the patient’s medical history with a disease-free interval of less than three years (except for previously treated basal cell carcinoma and in situ carcinoma of the uterine cervix);

(9.) Any contraindication, in the opinion of the blood transfusionist, to undergo leukapheresis (*e.g*. severe anaemia, thrombocytopenia, oral anticoagulant therapy).

### Clinical endpoint assessments

#### Tumor assessment

This study will use immune-related response criteria (irRC) [47], which is a further refinement of modified WHO (World Health Organization) criteria, to better describe tumor response in subjects undergoing immunotherapy (Table 1). irRC were created because of the natural history of clinical response observed in subjects treated with immunological agents such as ipilimumab, which differ from those experienced by individuals receiving other classes of anti-cancer agents.

**Table 1:** **Immune**-**response response criteria** (**irRC**)

**Index lesions**	**Non-index lesions**	**New measurable lesions**	**New non**-**measurable lesions**	**% Change in tumor burden**	**irRC-overall response**
CR	CR	No	No	−100%	irCR
PR	Any	Any	Any	≥ -50%	irPR
PR	Any	Any	Any	< -50% to < +25%	irSD
PR	Any	Any	Any	≥ +25%	irPD
SD	Any	Any	Any	< -50% to < +25%	irSD
SD	Any	Any	Any	≥ +25%	irPD
PD	Any	Any	Any	≥ +25%	irPD
SD	Any	Any	Any	< -50% to < +25%	irSD
SD	Any	Any	Any	≥ +25%	irPD
PD	Any	Any	Any	≥ +25%	irPD

*irRC*: immune-related response criteria; *PR*: partial response; *SD*: stable disease; *PD*: progressive disease.

The main differences between irRC criteria and conventional criteria are:

– new measurable lesions are incorporated into the tumor burden (*e.g*. added to the index lesions) and do not define progression unless the total measurable tumor burden increases by the required amount of 25%;

– new non-measurable lesions (including bone lesions) are not considered progression if the total measurable tumor burden is stable or shrinking;

– changes in non-measurable lesions only contribute to the definition of irCR;

– disease progression must be confirmed.

### Immunologic endpoint assessment

#### Immunologic efficacy

Immunologic efficacy, *i.e*. the ability of the different treatments to efficiently induce an anti-tumor immune response, will be measured by the DTH test against ATL and KLH after at least four vaccine induction doses. DTH testing will be performed in all patients on day −1 (*pre*-*treatment DTH*, which showed no reactivity in the vast majority of patients evaluated in our previous studies), and one day before the 5th, 8th, 11th and 14th vaccine dose (*post*-*treatment DTH*). Scalar doses (100, 50, 20, 10 and 5 μg) of autologous tumor lysate and KLH (pulsed into DC together with tumor lysate as an adjuvant) will be used. The diameter of the induration/erythema observed after 24 hours will be recorded according to the following scale: 0–5 mm grade 1, 6–10 mm grade 2, 11–20 mm grade 3, >21 mm grade 4. As DTH reactivity to lower concentrations of the antigen (s) is closely related to more intense antigen-specific immune response, the score results will be normalized against the concentration itself and transformed into a 0–80 scale for analysis purposes.

The best result obtained for each patient at any of the post-treatment DTHs, either for ATL or KLH, will be taken into account for data analysis (best normalized score). The best normalized score distributions across the different treatment arms will be evaluated according to non-parametric tests and, if the differences are found to be statistically significant, the specific primary objective will be considered fulfilled.

#### ELISPOT analysis

IFN-γ-ELISPOT assay will be performed using a commercial kit (Tema Ricerca, Bologna, Italy) according to the manufacturer’s instructions. Total PBMCs (10^5^ cells/well) will be used as responding cells and stimulated with 1 μg/mL of peptide covering the entire set of tumor antigens known to be expressed in melanoma (tyrosinase, MART1, MAGEA3, MAGEA1, gp100, NY-ESO, SOX2 and survivin; JPT GmbH, Germany). Plates will be evaluated by a computer-assisted ELISPOT reader (Eli.Expert, A.EL.VIS, Hannover, Germany). The patients’ DTH scores will also be evaluated in combination with IFN-γ-ELISPOT analysis of tumor antigen-specific circulating effectors in the different treatment arms with the same time schedule. In particular, if no significant difference across the treatment arms is found for DTH testing, the distribution of the fold increase (*i.e*. the post-vaccine: pre-vaccine ratio values) in Spot Forming Cells (SFC)/100,000 total PBMC recorded after at least four vaccine doses compared to pre-vaccine values, obtained by ELISPOT analysis and after subtraction of SFC observed in non-stimulated PBMC, will be evaluated using the Student’s t-test across the different treatment arms. If differences are found to be statistically significant (significance level 0.05), the specific primary objective will be considered fulfilled.

### Assessment of the effects of preleukapheresis IFN-α on vaccine yield

The total number of vital ATL-loaded DC obtained from each leukapheresis will be measured by vital dye staining with trypan blue and microscopic counting after *in vitro* differentiation and maturation, according to the specific SOP currently adopted by the STC Lab. The value recorded will then be normalized to the volume of blood processed by leukapheresis (expressed in millilitres) to obtain the number of vital DC/ml of in processed blood. The distribution of vital DC number/ml of blood processed by leukapheresis observed in the different treatment arms (IFN-α *vs* no-IFN-α) will then be evaluated using the Student’s t-test and, if differences are found to be statistically significant, the specific primary objective will be considered fulfilled. Additional leukaphereses performed due to vaccine shortage will not be included in the analysis.

### Assessment of the effects of preleukapheresis IFN-α on DC potency

#### Immunologic potency analysis

The co-stimulatory ability of the vaccine will be assessed with a modified version of the COSTIM assay [48] utilizing IFN-γ as a read-out system which has been used in our laboratory to assess the equivalence of freshly-made *vs* cryopreserved vaccine (data not shown). This GMP-validated method relies on the capacity of co-stimulatory signals provided by DC, *per se* unable to fully activate responder T cells, to trigger a proliferative response or cytokine secretion in allogeneic T cells stimulated with substimulating doses (0.005 mg/ml) of the anti-CD3 antibody OKT3. Briefly, 1×10^5^ reference T cells are co-cultured in quadruplicate with DC in IFN-γ ELISPOT plates for 24 hours at a T:DC ratio of 10:1, in the presence of 0.005 mg/ml OKT3. T cells cultured in the presence of 0.005 mg/ml OKT3 or DC are used as negative control. To ensure intra- and inter-assay reproducibility, reference T cells from at least 3 healthy donors are used for each determination. After a 24-hour incubation at 37°C in 5% CO2, plates are developed according to the manufacturer’s instructions and evaluated by a computer-assisted ELISPOT reader (Eli.Expert, A.EL.VIS GmbH). The distribution of SFC numbers across the different treatment arms (IFN-α *vs* no-IFN-α) will be evaluated according to the Student’s t-test and, if the differences are found to be statistically significant, the specific primary objective will be considered fulfilled. For this endpoint, only the first leukapheresis will be included in the analysis.

#### Assessment of the effects of preleukapheresis IFN-α on TEM-8 expression

We demonstrated in previous works that patients who show upregulation of TEM-8 in DC after maturation (as assessed by flow cytometry and real-time PCR) do not develop anti-tumor immunity after DC vaccination. In order to assess this specific objective, total RNA will be extracted from immature and mature DC (5 × 10^6^ cells) and reverse transcribed to cDNA. Quantitative real-time PCR will be then performed using primer sets that cover all human TEM-8 splicing variants; appropriate housekeeping genes will also be amplified as normalization controls. The ratio between TEM-8 mRNA expression (normalized against housekeeping genes) in monocytes and in mDC will then be calculated. The distribution of the TEM-8 mRNA iDC:mDC ratio observed in the different treatment arms (IFN-α *vs* no-IFN-α) will be then evaluated using the Student’s t-test. If the differences are found to be statistically significant, the specific primary objective will be considered fulfilled.

### Sample size

This is a “randomized selection design” study based on the assumption that immunotherapy is expected to be effective only in patients showing efficient induction of antitumor immune responses (“targeted endpoint”) [49]. The Steinberg and Venzon approach permits the researcher to select one among a number of different treatment arms as being worthy of further evaluation in a subsequent study. This method requires an adequate gap in the number of immune responses among different arms be observed in order to limit the probability that the selected arm is, in actual fact, inferior to the others. Assuming an error probability of selecting an inferior arm of 10%, a gap of 2 patients with an immune response out of a total of 6 enrolled patients per arm is sufficient, regardless of the proportion of irOR expected in each arm, to show that the difference between the highest probability of response and the best response of the remaining arms is 15%. If the gap is inferior to 2 patients, no treatment arm can be considered better than another and new immunostimulating therapies will have to be identified to combine with the DC vaccine.

## Discussion

The recent development of targeted agents and new drugs has partially changed the clinical course of advanced melanoma patients. Toxicities from BRAF inhibitors or anti-CTLA-4 antibodies for melanoma, albeit low grade, are continuous and chronically impact on quality of life. Furthermore, some toxicities with an initially mild presentation can progress, if not rapidly recognized, into severe and potentially life-threatening events [50,51]. Conversely, the use of vaccines in melanoma patients has been historically associated with negligible toxicity, although clinical results are disappointing and somewhat contrasting. However, encouraging results have been reported in several studies of vaccination with dendritic cells, albeit obtained on small series of patients.

In this context we recently reported the results from a phase II clinical study of DC-based vaccine in metastatic melanoma in which 55.5% of evaluable patients showed a clinical benefit (PR + SD), with very low toxicity [15,16]. Like us [16,18], other groups [17] also observed that, patients who developed anti-tumor immunity after vaccination experienced a better clinical outcome. In particular, we saw that individuals developing delayed type hypersensitivity (DTH) against autologous tumor lysate or KLH after at least four courses of the vaccine showed a median OS of 22.9 months compared to 4.8 months for DTH-negative cases (Log-rank test, p = 0.007) [16].

These results prompted us to use combination treatments aimed at improving the rate of patients showing positive immunization after vaccination. In particular, we observed that all those who underwent therapy with low-dose IFN-α in the month before starting DC vaccination (unpublished data) showed positive immunisation after the first induction doses, suggesting that IFN-α priming, performed *in vivo* before leukapheresis, may enhance the immunostimulatory profile of DC. Moreover, IFN-α priming may also have a “mobilizing” activity on DC precursors: it was recently been reported that 1–3 MU subcutaneous IFN-α enhances the proportion of circulating CD14+ and CD14 + CD16+ monocytes in both healthy donors and melanoma patients [26]. On this basis, the administration of IFN-α before leukapheresis may positively modulate the immunological and clinical efficacy of DC vaccination. In addition to the potential for preleukapheresis IFN-α to improve the immunological efficacy of monocyte-derived DC vaccine, a synergism between immunotherapy and radiotherapy (the abscopal effect) has hypothesized in which radiotherapy “immunizes” the patient against cancer cells, converting the irradiated tissue into an *in situ vaccine*. Although, in some cases, radiotherapy alone can induce immunological memory protection, in others it loses its initial efficacy when recurrence occurs. Overall, findings from the literature suggest that radiotherapy may also act as an “immune response modifier” which can correct the immunosuppressive network created by tumor cells. In that respect, radiotherapy would seem to be capable of recovering the cancer-specific immune response after failure of immunotherapy, of boosting the naturally occurring anti-tumor immune response, and cooperating with cancer vaccines to facilitate the priming of a non-immunodominant response [52,53].

We designed a randomized, proof-of-principal study in which preleukapheresis radiotherapy and/or IFN-α are added to the DC vaccine with the aim of enhancing the vaccine-induced tumor immune response and, in doing so, of potentially improving survival. Moreover, these combination therapies should, in theory, maintain the same low toxicity profile observed in our previous studies. Dose and fraction of radiotherapy and the correct sequencing of therapies will also be evaluated. The identification of an effective treatment regimen could be used in a phase III trial or be applied to clinical practice.

## Abbreviations

AE: Adverse event; ALT: Alanine aminotransferase; ANC: Absolute neutrophil count; ASAT: Aspartate aminotransferase; AR: Adverse reaction; ATL: Autologous tumor lysate; CBC: Complete blood count; CI: Confidence interval; CNS: Central nervous system; CTLA-4: Cytotoxic T-lymphocyte antigen 4; CTscan: Computed tomography; CTC: Common toxicity criteria; DC: Dendritic cells; DCR: Disease control rate; DFS: Disease free survival; DM: Ministerial Decree; DTH: Delayed type hypersensitivity; ECOG: (Eastern Cooperative Oncology Group, ECOG Scale) Performance status; GCP: Good Clinical Practice; GM-CSF: Granulocyte/Monocyte colony stimulating factor; GMP: Good Manifacturing Practice; iDC: immature dendritic cells; IFN-α: Interferon α; Il-1β: Interleukin-1β; IL2: Interleukin 2; IL4: Interleukin 4; IL6: Interleukin 6; IL8: Interleukin 8; IR: Ionizing radiations; irBOR: immune-related best overall response; irCR: immune-related complete response; irDCR: immune-related disease control rate; irDOR: immune-related duration of response; irORR: immune-related overall response rate; irPD: immune-related progressive disease; irPFS: immune-related progression-free survival; irPR: immune-related partial response; irRC: immuno-related response criteria; irSD: immune-related stable disease; irTTP: immune-related time to progression; irTTR: immune-related time to response; KLH: Keyhole lympet hemocyanin; MHC: Major histocompatibility complex; NK: Natural killer; OS: Overall survival; PGE2: prostaglandin E2; PD: Progressive disease; RECIST: Response Evaluation Criteria In Solid Tumors; RT: Ionizing radiation therapy; SD: Stable disease; SFC: Spot forming cells; SPD: Sum of the perpendicular diameters; TEM-8: Tumor endothelial marker-8; TNF-α: Tumor necrosis factor α; TPFS: Total progression-free survival; irTTP: Immune-related time to progression; WBC: White blood cells; WHO: World Health Organization.

## Competing interests

The authors declare that they have no competing interest.

## Authors' contributions

RR, LV, ON and MG designed and wrote the study protocol. LR and FdR are responsible for the clinical activity. GG is the Clinical Study Coordinator. MP, LF, AMG, VA, PA, VS, SC and AR are involved in the manufacture of the investigational product. EP and AR are responsible for performing the radiotherapy treatments. LT coordinates the collection of biological samples for the study’s biological endpoints. MG is the Principal Investigator of the study. All authors read and approved the final version of this manuscript.

## Supplementary Material

Additional file 1 Description of manufacturing process. Click here for file
