# Supplementary material for: Vaccination with autologous dendritic cells loaded with autologous tumor lysate or homogenate combined with immunomodulating radiotherapy and/or preleukapheresis IFN-α in patients with metastatic melanoma: a randomised “proof-of-principle” phase II study
Source: J Transl Med. 2014 Jul 22;12:209. doi: 10.1186/1479-5876-12-209 (PMC4223722; doi:10.1186/1479-5876-12-209)
Supplement: Additional file 1 — Description of manufacturing process. [file 1479-5876-12-209-S1.docx]

**DESCRIPTION OF MANUFACTURING PROCESS**

***Tumor homogenate preparation***

Patients eligible for treatment will undergo surgical resection of one or more tumor localizations and the tumor tissue obtained will be utilized for the preparation of the autologous tumor homogenate employed for dendritic cell pulsing. All patients will be tested for the presence of HIV, HBV, HCV and treponema infection as per Italian legislation (DL n.16, 25/01/2010) and admitted to the procedure only if negative for the above tests in the 30 days before surgery.

After acceptance, tumor tissue will be stored at –80° until product manufacturing. Tumor homogenate is ~~then~~ prepared from stored tumor tissue as follows: tumor tissue is cut into small pieces (max 4 mm), placed in a sterile saline solution (1:5 w/vol) in M tubes (Miltenyi), and homogenated with GentleMACS (Miltenyi) utilizing the program RNA02. After homogenization, the supernatant is filtered through 0.43 μ and 0.22 μ Millipore PVDF filters. Total protein content of the tumor homogenate is assessed by the Bradford method and tumor homogenate is aliquoted and stored at -80°C until utilization.

Tumor homogenate is definitively released upon:

1) histological diagnosis of melanoma on the resected tissue utilized for homogenate preparation;

2) homogenate sterility, assessed by culture examination;

3) bioburden lower than 10^5^ cfu/50 ml

***Vaccine manufacturing***

Patients eligible for the treatment will undergo apheresis to collect an amount of peripheral blood mononuclear cells (PBMC) sufficient to obtain at least 4/5 vaccine doses (mean processed blood volumes 5.7 l, mean PBMC collected 6.8 x 10^9^).

Briefly, 200 x 10^6^ PBMC are plated in T150 flasks (Corning) and non-adherent cells are removed after 2 hours. Adherent cells are then cultered for 6 days in CellGro DC (CellGenix) supplemented with 2000 UI/ml IL-4 and 2000 UI/ml GM-CSF to differentiate them into immature dendritic cells. Immature dendritic cells are then pulsed for 12-16 hours with autologous tumor homogenate (100 μg/ml).

Acceptance criteria for immature dendritic cells are the following:

− Cellular Vitality > 70%

− Cellular Purity > 60%

− HLA-DR+ cells > 60%

− CD86+ cells < 60%

− CD83+ cells < 20%

After pulsing, immature dendritic cells are then washed with CellGro DC (CellGenix) and matured for 48 hours in the same medium supplemented with 20 ng/ml TNF-α, 2000 IU/ml IL-6, 20 ng/ml IL1-β, and 1 μg/ml PGE2. After pulsing and maturation, dendritic cells are harvested, washed, counted, resuspended in autologous plasma, aliquoted, frozen by automated programmed freezing, and stored in nitrogen vapor until utilization. Mean recovery of mature dendritic cells obtained is 169.57x10^6^ (min 34.5, max 590 x 10^6^).

Dendritic cells are washed and resuspended in NaCl 0.9% just before administration.

Acceptance criteria for mature dendritic cells are the following:

− Cellular Vitality > 70%

− Cellular Purity > 60%

− HLA-DR+ cells > 60%

− CD80+ cells > 50%

− CD86+ cells > 60%

− CD83+ cells > 4 0%

- Endotoxin < 0.5 EU/ml

- Micoplasma: absent

The stability of dendritic cells stored within the syringe utilized for its administration was evaluated in 6 batches as follows.

Dendritic cells were stored at room temperature in the syringe and vitally counted at different times. Vitality, *i.e.* the percentage of vital/ total number of cells (dead + living), and purity (the percentage of mature dendritic cells/total living cells) were recorded and are reported in Table 1. Data analysis showed that the formulated product in syringe did not reach the critical time (T_c_), *i.e.* the time required to lower cellular product vitality under 70% and purity under 60%, after 120’.

| **Time** | **Average product vitality (range)** | **Average product purity (range)** |
| --- | --- | --- |
| 0’ | 91 (83-96) | 83 (64-90) |
| 15’ | 89 (75-93) | 81 (61-90) |
| 30’ | 87 (80-98) | 78 (61-91) |
| 45’ | 87 (80-93) | 84 (61-95) |
| 60’ | 88 (74-96) | 82 (64-94) |
| 75’ | 87 (84-93) | 88 (62-97) |
| 90’ | 84 (71-96) | 87 (61-96) |
| 105’ | 85 (73-97) | 85 (60-96) |
| 120’ | 81 (70-88) | 84 (61-98) |

Table 1. Analysis of product stability. Average values and range for the 6 batches analyzed are shown at the different times.
